# Inhibition of the checkpoint protein PD-1 by the therapeutic antibody pembrolizumab outlined by quantum chemistry

**DOI:** 10.1038/s41598-018-20325-0

**Published:** 2018-01-30

**Authors:** Ana Beatriz M. L. A. Tavares, José X. Lima Neto, Umberto L. Fulco, Eudenilson L. Albuquerque

**Affiliations:** Departamento de Biofísica e Farmacologia, Universidade Federal do Rio Grande do Norte, 59072-970 Natal, RN Brazil

## Abstract

Much of the recent excitement in the cancer immunotherapy approach has been generated by the recognition that immune checkpoint proteins, like the receptor PD-1, can be blocked by antibody-based drugs with profound effects. Promising clinical data have already been released pointing to the efficiency of the drug pembrolizumab to block the PD-1 pathway, triggering the T-lymphocytes to destroy the cancer cells. Thus, a deep understanding of this drug/receptor complex is essential for the improvement of new drugs targeting the protein PD-1. In this context, by employing quantum chemistry methods based on the Density Functional Theory (DFT), we investigate in silico the binding energy features of the receptor PD-1 in complex with its drug inhibitor. Our computational results give a better understanding of the binding mechanisms, being also an efficient alternative towards the development of antibody-based drugs, pointing to new treatments for cancer therapy.

## Introduction

The World Health Organization (WHO) labels cancer as one of the most pressing health challenges in the world. Statistical data from the National Cancer Institute (NCI) show that one in two (three) men (women) will develop this disease. Cancer is a leading cause of death, accounting for over 8.8 million casualties and 14.1 million cases diagnosed worldwide in 2015, numbers that are expected to increase in the coming years^1^. It is characterized by an uncontrolled growth of cells in the body, with the potential to invade or spread to its other parts with the formation of metastases. It is induced by mutations in the genome of a cell population, which changes the normal function of various classes of protein families, such as cytokines, cell surface receptors, signal transducers and transcription factor, making it one of the most difficult and complex disease to treat^2–4^. The more widely the cancer spreads, the harder it becomes to eradicate. In 2013, oncology was ranked on the top therapeutic class by worldwide sales, amounting to $73 billion^5^. However, though highly expensive, the treatment suffers several fails due to a defense mechanisms developed by some malignant cells, mainly those related to the activation of drug resistance processes^6^.

Although these mutations provide a selective vantage to populations of cancer cells, they also increase their divergences from the normal one, which can allow the recognition by the immune system cells, such as the T lymphocytes (T cells) and B cells^7^. Nevertheless, tumors also evolved to deceive immune cells, including the ability to activate co-inhibitory signaling pathways on T cells by immune checkpoint proteins, such as the cytotoxic T-lymphocite protein 4 (CTLA-4) and the programmed cell death protein 1 (PD-1), leading to a state of immune tolerance^8,9^.

The molecular identification of cancer antigens helped the creation of new approaches for effective therapies, giving rise to a new era of treatment in which our own immune system evade the block created by malignant cells and fights against them. Among these new treatments, the immune checkpoint therapy has been clinically validated as an effective treatment for many cancer types^10^. The blockade of the ligand-receptor interaction of these immune checkpoint molecules can directly increase the function of the T-cells, whereas it has been shown as a way to release the immune system to unleash anti-tumor immune response^11^.

The receptor PD-1 (*Pdcd1* gene on chromossome 2) is an immune cell-specific surface inhibitor, mainly expressed in the late effector phase on activated CD4^+^/CD8^+^ T cells, B cells, monocytes, natural killer T cells, and antigen-presenting cells (APC), including dendritic cells^12–14^. It is a transmembrane glycoprotein of the Ig superfamily, accounting for 288 amino-acids, displaying four domains including a single V-set immunoglobulin superfamily (IgSF) domain, a stalk, a transmembrane domain and a cytoplasmic domain, which contains two tyrosine-based immunoreceptor signaling motifs: the inhibitory motif (ITIM) and the switch one (ITSM)^15,16^. Due to its relevance for immune system maintenance, a number of crystallographic structures have been published related to the human extracellular domain of PD-1, in apo form or bound to several ligands, making possible to recognize its overall structural architecture^17–19^. A comparison among them allow us to describe the PD-1 extracellular domain in a canonical IgV-type topology containing a *β*-sandwich arrangement formed by front and back *β*-strand faces of interacting domains, GFCC’ and ABED *β*-sheets, respectively (for a review see ref.^20^).

Together with its ligants PD-L1/PD-L2, the receptor PD1 form a family of immune checkpoint proteins that act as co-inhibitory factors, which can halt or limit the development of the T cell response. Through interactions with them, the protein PD-1 affects negatively the function of the T and B cells by inhibiting their immune check, ensuring that the immune system is activated only at the appropriate time in order to minimize the possibility of chronic autoimmune inflammation, leading to a decrease of the cytokine production and antibody formation. On the other hand, tumor cells exploit this immune-checkpoint pathway as a mechanism to evade detection and inhibit the immune response, preventing the immune system from killing cancer cells. Many types of cells can express PD-L1, including tumor cells and immune cells after exposure to cytokines, while PD-L2 is expressed mainly on dendritic cells in normal tissues, making the PD-1/PD-L1 interaction more suitable to cancer therapies^21^. Thus, the search for antibodies that block the interaction between the receptor PD-1 and its ligands has been seen as a major therapeutic advancement in oncology, once it shows response often durable without causing serious toxicity effects in most people^22^.

Currently, a number of cancer immunotherapy agents that target the PD-1 receptor have been developed. Among them, two antibody-based agents targeting PD-1 were approved by the US Food and Drug Administration (US-FDA) and other agencies around the world, namely the nivolumab and pembrolizumab drugs. Pembrolizumab is a humanized monoclonal antibody, belonging to the immunoglobulin IgG4 subclass with potential immune checkpoint inhibitory and antineoplastic activities. Upon administration, it blocks the interaction between the programmed cell death protein PD-1 on T cells with its ligands which results in the activation of T-cell-mediated immune responses against tumor cells^23^. It was the first anti-PD-1 agent to be approved by the FDA, being nowadays used to treat some cancer types where cells express PD-L1, including advanced melanoma, advanced non-small cell lung cancer (NSCLC), recurrent or metastatic head and neck squamous cell carcinoma, classical Hodgkin lymphoma (cHL), and counting, the list being increasing with the release of new clinical trials^24^.

Although these antibodies are associated with substantial benefits, the immune checkpoint blockade can lead to inflammatory side-effects. Besides, there is a necessity for new drugs targeting PD-1^24,25^. Thus, obtain a deep understanding of the human PD-1/therapeutic antibody complex is essential for our knowledge about its inhibition mechanism and the design of improved anti-PD-1 therapeutics. In this sense, it has been shown that patients making use of pembrolizumab has presented better results and fewer side-effects than those using the nivolumab drug, turning the former a suitable candidate to provide new compounds based on its structure.

**Figure 1:**
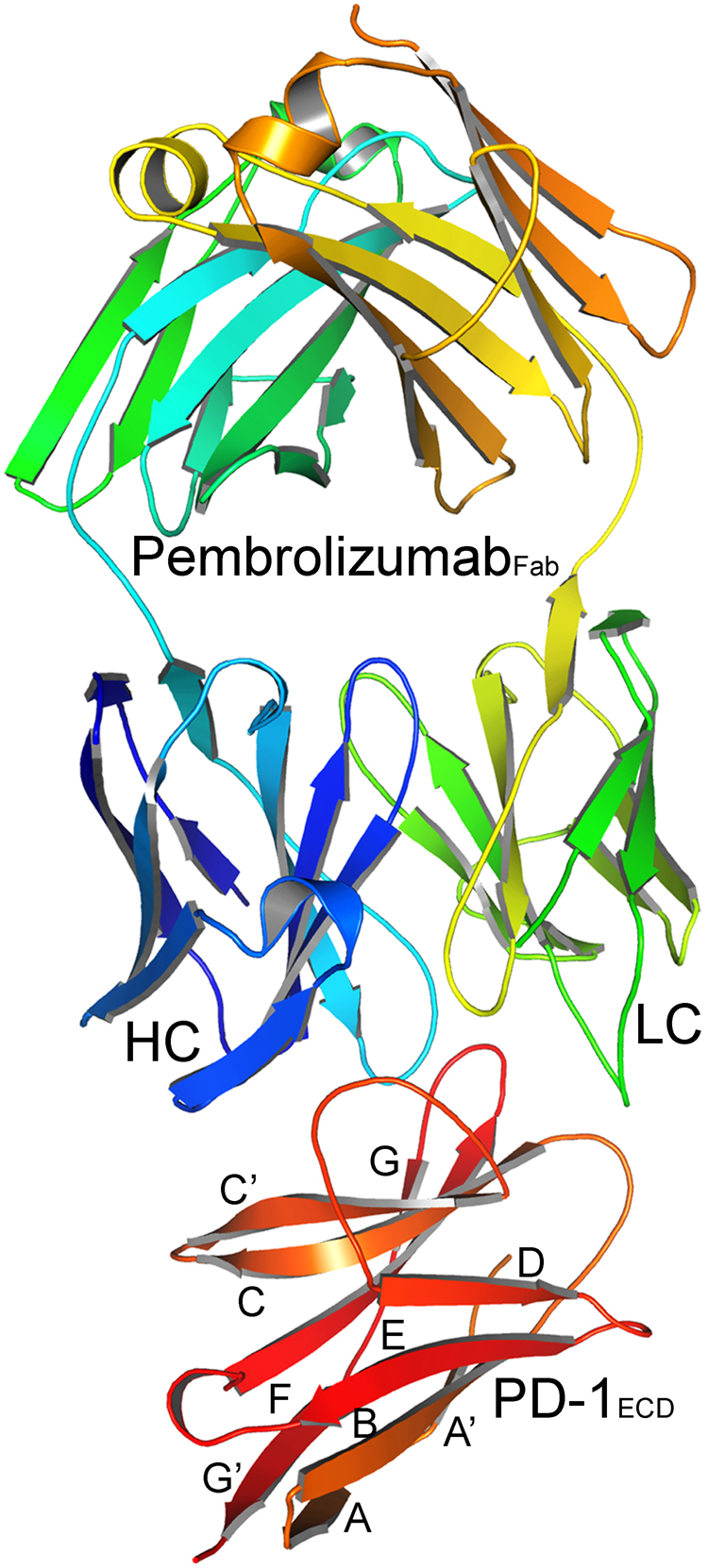
Structural representation of the antigen-binding fragment (Fab) of pembrolizumab (PDB ID:5GGS) in complex with the extracellular domain of human PD-1 receptor. Here, HC and LC means heavy-chain and light-chain, respectively.

To push this field forward, we intend here to investigate the pembrolizumab recognition surface on PD-1 under a quantum mechanical-based point of view. Our main goal is the supply of enough information at the binding interface that substantially contribute to the affinity and specificity between the receptor (PD-1) and the therapeutic antibody (pembrolizumab). For this task, we take into account the crystallographic structure of the antigen-binding fragment (Fab) of pembrolizumab in complex with the extracellular domain of human PD-1 (PD-1_*ECD*_) determined at a resolution of 2.00 Å^26^, as depicted in Fig. 1, with the structural segments of the receptor PD-1 labeled with different letters. Afterwards, it is submitted to a fragmentation scheme based on the molecular fractionation with conjugate caps (MFCC) method^27,28^. Our results are the first attempt to outline the pembrolizumab/PD-1 interaction based in quantum mechanics, given an individual residue-residue interaction energy, which could be useful for the development of new pharmaceutical drugs targeting PD-1, and a key tool to avoid the onset of a number of cancerous tumors.

## Results

The programmed cell death protein 1 (PD-1) is an important regulator for the immune tolerance and T cell exhaustion, being recently emerged as a key target in the treatment of several types of cancer. It is expressed after the T cell activation and binds to the ligants PD-L1 and PD-L2, suppressing immune response against autoantigens and playing an important role in the maintenance of peripheral immune tolerance^13^. However, the ligant PD-L1 is often overexpressed in different tumor including lymphoma, melanoma, non-small-cell lung cancer and other types, making the PD-1/PD-L1 signaling pathway crucial in dampening the immune surveillance of the tumor^29^.

**Figure 2:**
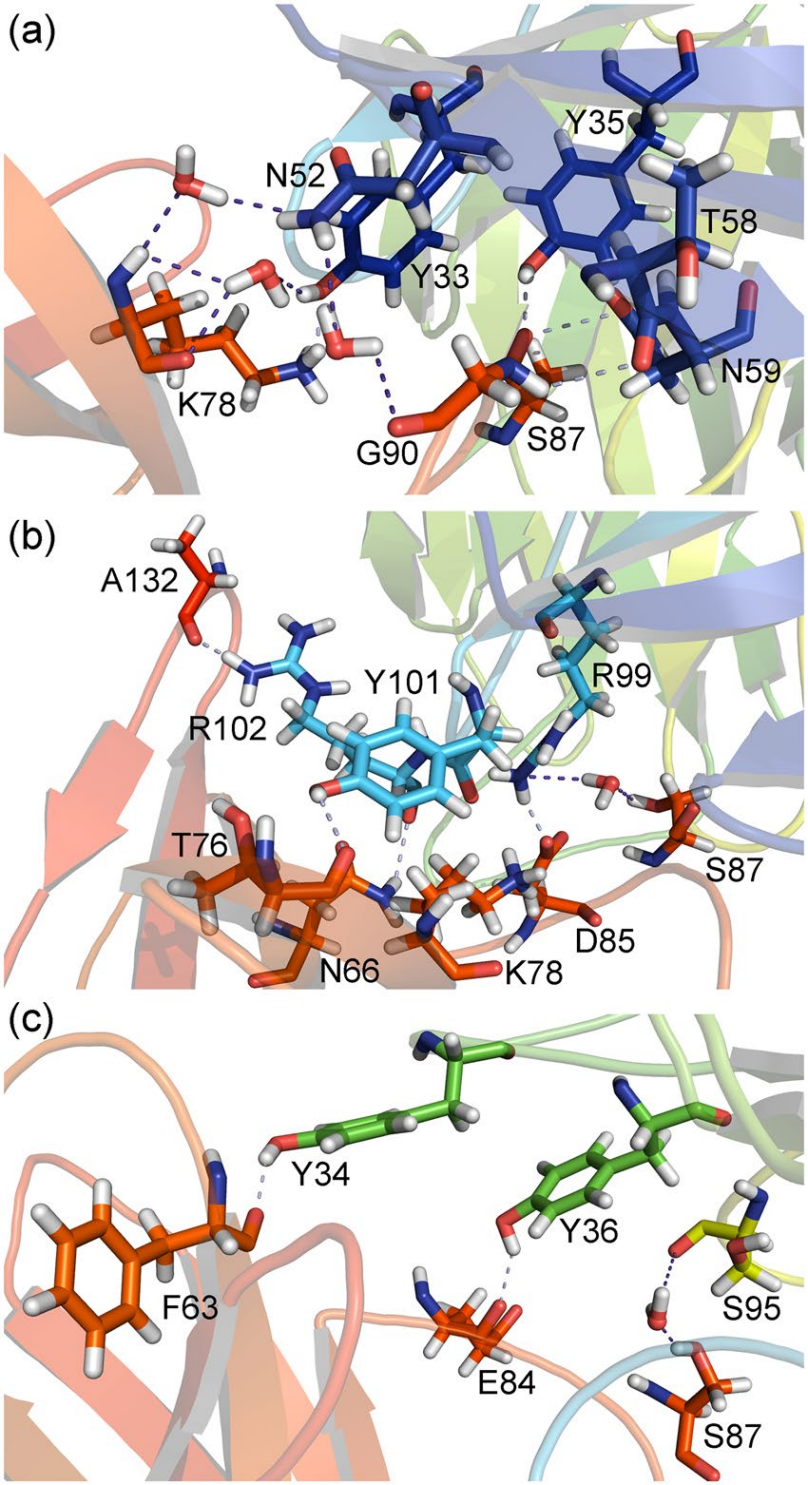
Interaction patterns of pembrolizumab/PD-1 recognition surface involving the amino-acid residues and the water molecules. (**a**) and (**b**) heavy-chain (HC) residues; (**c**) light-chain (LC) residues.

In this context, the target of the PD-1/PD-L1 interaction with monoclonal antibodies has demonstrated to be an important strategy for the control and eradication of several types of cancers. The treatment with pembrolizumab was approved by US-FDA in 2014 for advanced melanoma, and recently it has also been approved to an increasing number of cancer types, such as Hodgkin lymphoma and non-small-cell lung cancer. It is believed that the weak frequency in humans and the induction of cell activation, characteristics of the members of IgG4 subclass, are some of the main basis for the success of this therapeutic compound^23^. As we can see from Fig. 2, the pembrolizumab recognition surface on PD-1 is filled by intermolecular direct and water-mediated hydrogen bonds (HBs and wHBs, respectively), non-conventional hydrogen bonds (nHBs), salt bridges (SB) and hydrophobic contacts. Therefore, mapping the relevant interactions among the pembrolizumab/PD-1 surface complex is highly important to the rationale drug development of new compounds.

In this work, we employed the MFCC/DFT scheme to assess the relative energetic contribution of the individual as well as pair-interaction contribution of each residue from the pembrolizumab/PD-1 complex. From now on we will describe all the steps in this recognition process.

### Pembrolizumab/PD-1 recognition surface

**Figure 3:**
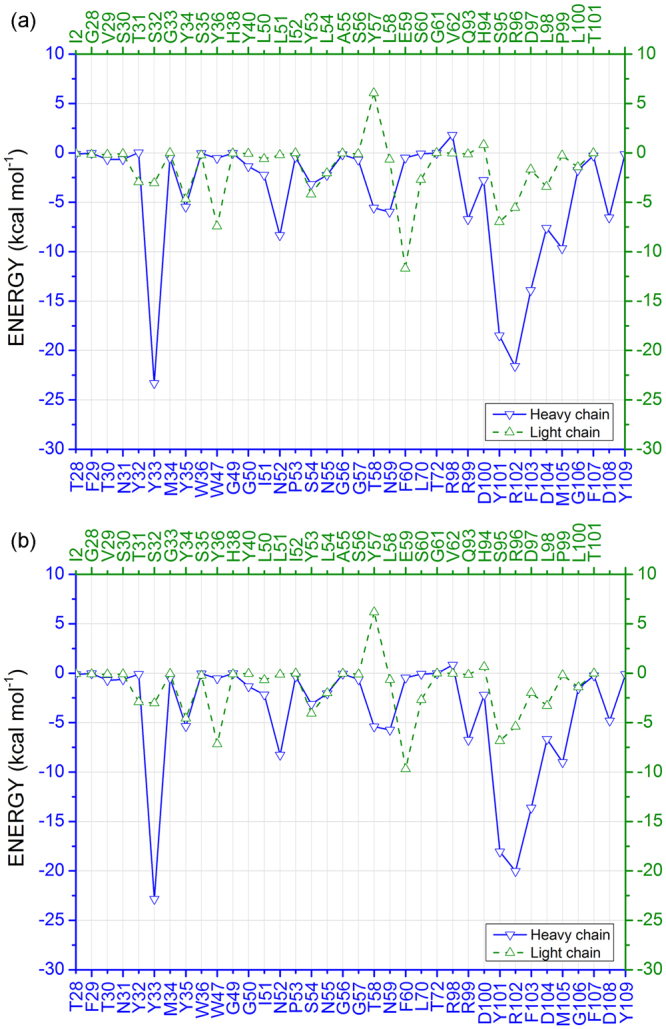
Energy profile for each pembrolizumab amino-acid residues in recognition surface. (**a**) This figure represents the sum of interaction energies of the pembrolizumab residues with each amino-acid from PD-1 within a radius of 8.0 Å, using the dielectric constant *ε*_20_. (**b**) The same for *ε*_40_. Blue solid line represents the heavy-chain energy spectrum, while the green dashed line is used to depict the light-chain one.

Through a structural analysis of the binding site, 408 pair of interactions were detected within a range of 8.0 Å. Figure 3 shows the sum of all interaction energies from each individual residue of the pembrolizumab drug (R _*i*_), taking into account a dielectric constant *ε* = 20 (*ε*_20_) and 40 (*ε*_40_) depicted in Fig. 3a and b, respectively. One can note that only two residues (one residue) in the pembrolizumab heavy-chain (HC) energy spectrum are (is) repelled in *ε* = 20 (*ε* = 40), while four (three) residues are repelled in its light-chain (LC) one, namely: *Y*32_*HC*_ (*ε*_20_: 0.04 kcal mol^−1^; *ε*_40_: −0.10 kcal mol^−1^), *R*98_*HC*_ (*ε*_20_: 1.80 kcal mol^−1^; *ε*_40_: 0.85 kcal mol^−1^), *G*33_*LC*_ (*ε*_20_: 0.008 kcal mol^−1^; *ε*_40_: −0.04 kcal mol^−1^), *Y*57_*LC*_ (*ε*_20_: 6.06 kcal mol^−1^; *ε*_40_: 6.17 kcal mol^−1^), *G*61_*LC*_ (*ε*_20_: 0.01 kcal mol^−1^; *ε*_40_: −0.002 kcal mol^−1^) and *H*94_*LC*_ (*ε*_20_: 0.82 kcal mol^−1^; *ε*_40_: 0.64 kcal mol^−1^). For instance, the energy value 0.04 kcal mol^−1^ calculated for *Y*32 (HC and LC designated residues, respectively) represents the sum of the binding energies of the four amino-acids that compose the receptor PD-1 and are within a radius distance of 8.0 Å from it, namely: *K*78, *P*89, *K*131 and *A*132. Thus, one can see that the repulsive energies are almost insignificant, excluding the residue *Y*57 that shows the highest one for both dielectric constant values *ε*_20_ and *ε*_40_.

All other residues shown attractive interactions (negative energies), with the most intense one being observed for the heavy-chain amino-acids *Y*33_*HC*_ (*ε*_20_: −23.32 kcal mol^−1^; *ε*_40_: −22.84 kcal mol^−1^), *Y*101_*HC*_ (*ε*_20_: −18.49 kcal mol^−1^; *ε*_40_: −18.07 kcal mol^−1^), *R*102_*HC*_ (*ε*_20_: −21.58 kcal mol^−1^; *ε*_40_: −20.03 kcal mol^−1^) and *F*103_*HC*_ (*ε*_20_: −13.91 kcal mol^−1^; *ε*_40_: −13.62 kcal mol^−1^), and light-chain residues *Y*36_*LC*_ (*ε*_20_: −7.41 mol^−1^; *ε*_40_: −7.16 kcal mol^−1^), *E*59_*LC*_ (*ε*_20_: −11.69 kcal mol^−1^; *ε*_40_: −9.69 kcal mol^−1^) and *S*95_*LC*_ (*ε*_20_: −6.99 kcal mol^−1^; *ε*_40_: −6.85 kcal mol^−1^).

### Pembrolizumab heavy-chain/PD-1 receptor interaction energy

Among the 408 residue-residue pairs analyzed here, 260 were pembrolizumab heavy-chain/PD-1 interactions. It is a reflection of the proximity between the HC/LC pembrolizumab with the receptor PD-1 represented in crystallographic structures, where pembrolizumab _*Fab*_ heavy-chain fragment is closer to the PD-1 receptor than the light-chain one^26,30^. Besides a higher number of pairs, the sum of the energetic interaction between the pembrolizumab heavy-chain and the PD-1 receptor amino-acids shows also the higher value, accounting for -142.50 kcal mol^−1^ (−138.33 kcal mol^−1^) for the dielectric constant *ε*_20_ (*ε*_40_).

**Figure 4:**
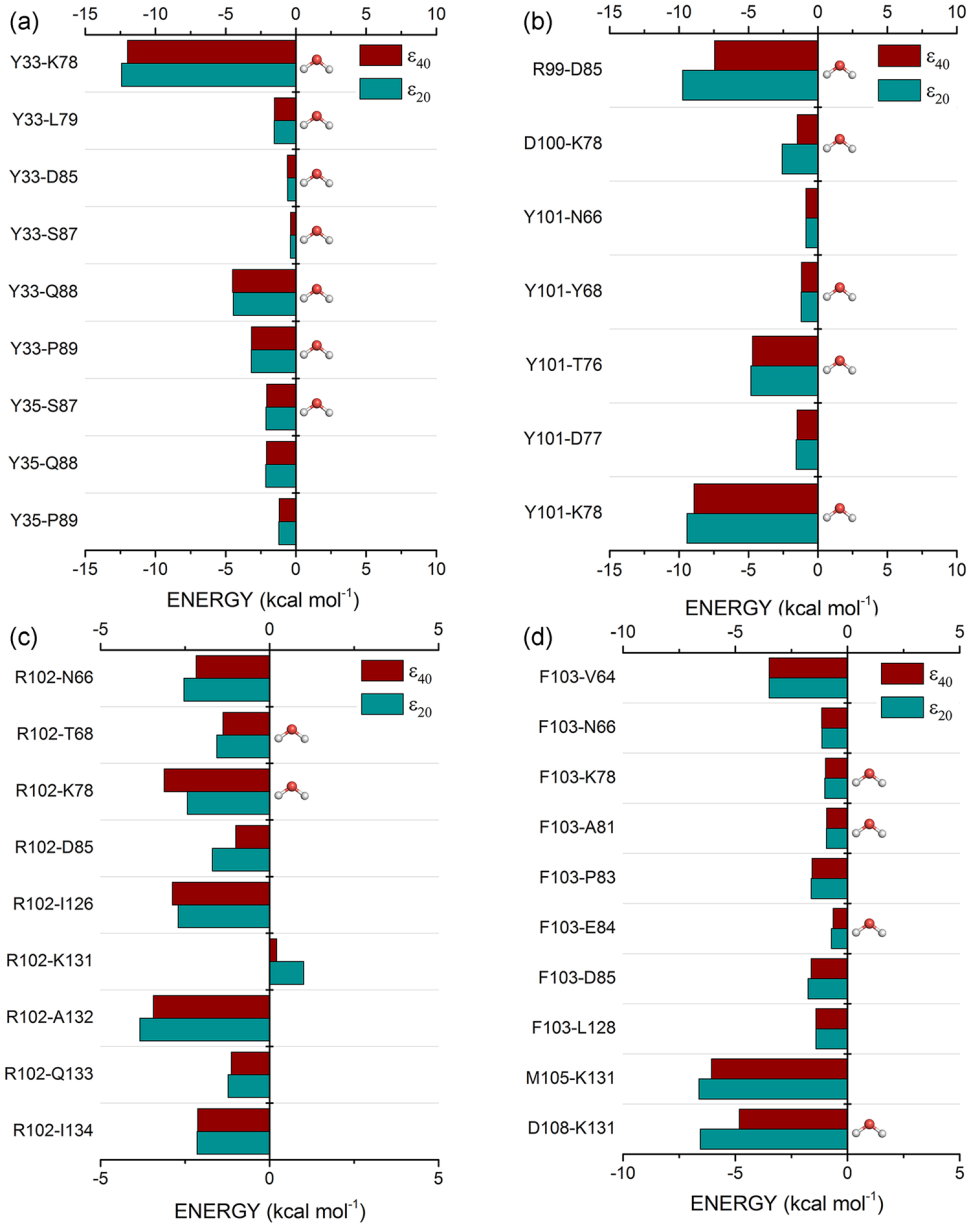
Graphical panel presenting the most relevant interactions involving the pembrolizumab heavy-chain residues. (**a**) *Y*33 and *Y*35; (**b**) *R*99, *D*100 and *Y*101; (**c**) *R*102 and (**d**) *F*103, *M*105 and *D*108, respectively.

Although *Y*33_*HC*_ has been shown to be the most energetic pembrolizumab amino-acid residue, it only interacts with 15 residues from the PD-1 receptor. Figure 4a depicts the highest interaction energies calculated to *Y*33_*HC*_/PD-1 residues. As one can see, the strongest energy of *Y*33_*HC*_ is mainly related to its binding with three residues: *K*78 (*ε*_20_: −12.42 kcal mol^−1^; *ε*_40_: −11.99 kcal mol^−1^), *Q*88 (*ε*_20_: −4.45 kcal mol^−1^; *ε*_40_: −4.51 kcal mol^−1^) and *P*89 (*ε*_20_: −3.17 kcal mol^−1^; *ε*_40_: −3.15 kcal mol^−1^). Meanwhile, *Y*101_*HC*_, *R*102_*HC*_ and *Y*103_*HC*_ are involved with 23, 30 and 21 pairs, respectively. As shown in Fig. 4b, *Y*101_*HC*_ has its most intense interaction with the residue *K*78 (*ε*_20_: −9.43 kcal mol^−1^; *ε*_40_: −8.92 kcal mol^−1^), being followed by the residue *T*76 (*ε*_20_: −4.82 kcal mol^−1^; *ε*_40_: −4.71 kcal mol^−1^). The residue *R*102_*HC*_ does not show a strong individual interaction with none of the PD-1 residues (Fig. 4c), its high binding energy being due to a number of minor energies contributions, including those from the residues *A*132 (*ε*_20_: −3.84 kcal mol^−1^; *ε*_40_: −3.44 kcal mol^−1^), *I*126 (*ε*_20_: −2.71 kcal mol^−1^; *ε*_40_: −2.87 kcal mol^−1^), *K*78 (*ε*_20_: −2.43 kcal mol^−1^; *ε*_40_: −3.12 kcal mol^−1^) and *N*66 (*ε*_20_: −2.53 kcal mol^−1^; *ε*_40_: −2.17 kcal mol^−1^). Similar to *R*102, *Y*103_*HC*_ binding energy is composed by the sum of a number of small interactions (Fig. 4d), with the strongest being associated with the residue *V*64 (*ε*_20_: −3.49 kcal mol^−1^; *ε*_40_: −3.49 kcal mol^−1^).

**Figure 5:**
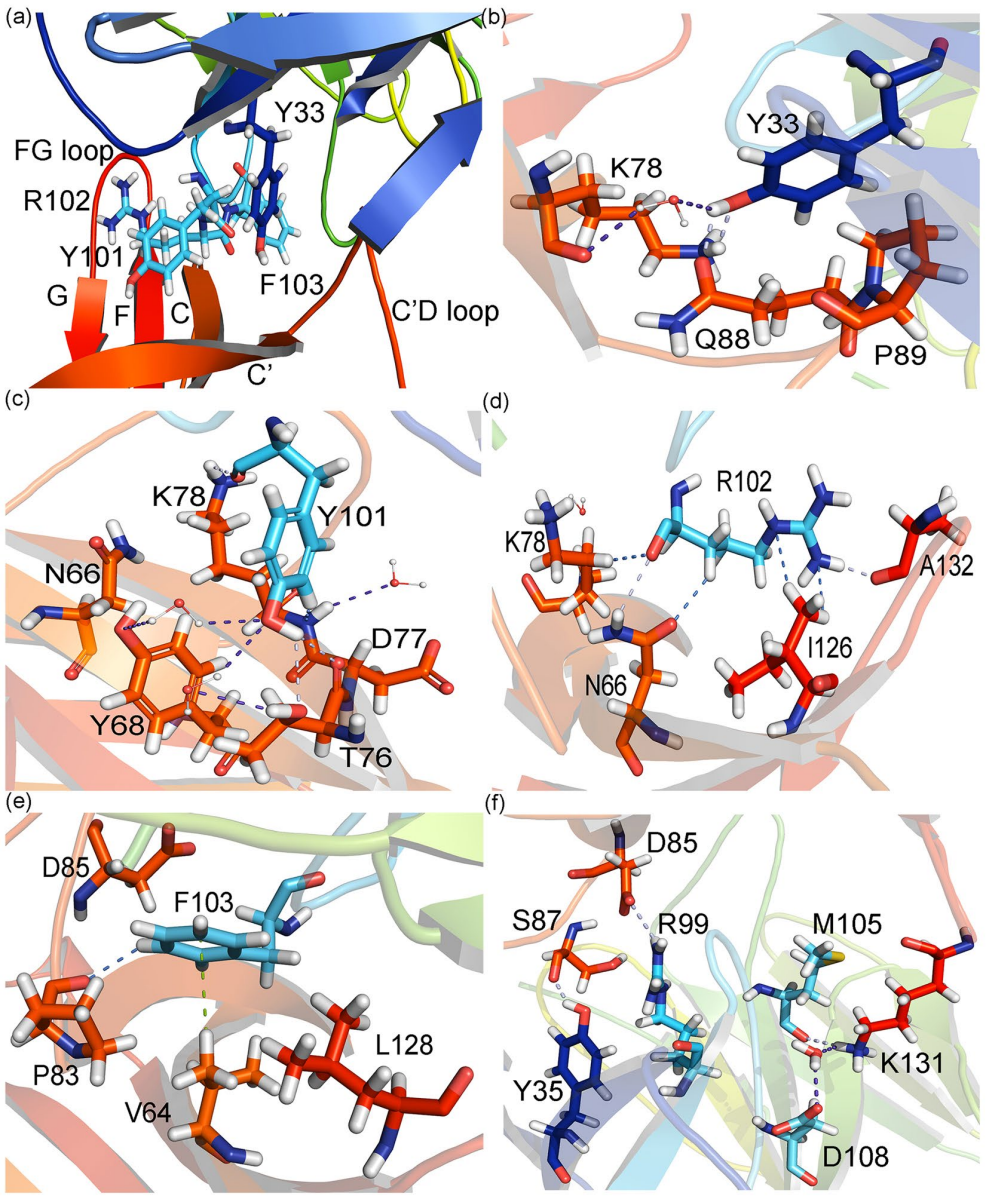
Intermolecular interactions of the most energetic pembrolizumab heavy-chain residues. (**a**) Structural representation of the residues *Y*33_*HC*_, *Y*101_*HC*_, *R*102_*HC*_ and *F*103_*HC*_ within the PD-1 binding site. (**b**)–(**f**) Interaction of these amino-acids with the most relevant residues of the receptor PD-1, respectively. Dashed lines in light blue (purple) (marine) represent direct (water-mediated) (non-conventional) hydrogen bonds, and the green lines represent *σ*-*π* interactions.

Figure 5a displays how close the residues *Y*33_*HC*_, *Y*101_*HC*_, *R*102_*HC*_ and *Y*103_*HC*_ are to the PD-1 receptor. Analyzing it one can understand the reason why these amino-acids present some of the largest number of pair-interaction and binding energies. All of them are involved in a network of hydrogen bonds with the PD-1 residues from CC’FG *β*-strands and some of its loops, mainly those belonging to the C’D loop, which was described to intrude into the complementary determining region (CDR) of the pembrolizumab drug, a variable portion present in some igG molecules responsible for the recognition of specific antigens^30^. Figure 5b depicts some of these interactions, starting with the residue *Y*33_*HC*_. Here one can see that it forms two direct hydrogen bonds with the residues *K*78 charged amine group and *Q*88 side-chain oxygen. Besides, the residue *Y*33_*HC*_ also makes a water-mediated hydrogen bond with the *K*78 carbonyl group of the main-chain, and is involved in non-polar contacts with *P*89. The two hydrogen bonds between the residues *Y*33_*HC*_-*K*78 give the major contribution to their high energy.

The residues *Y*101_*HC*_, *R*102_*HC*_ and *Y*103_*HC*_ interaction network is shown in Fig. 5c–e. Figure 5c depicts 3 directs, 2 water-mediated and 1 non-conventional hydrogen bonds. The strongest interaction of this residue is related to the pair *Y*101_*HC*_-*K*78, where it creates a direct hydrogen bond with charged side-chain amine and a non-conventional hydrogen bond with amine of the main-chain. Three hydrogen bonds are formed with the residue *T*76, two with its oxygen atoms (carbonyl and hydroxyl) and a water-mediated with the hydroxyl group. It is also involved in one wHB with the residue Y68 side-chain hydroxyl and in some non-polar contacts with the residues *D*77 and *N*66. The residues *R*102_*HC*_ and *Y*103_*HC*_ are mainly involved in weak interactions. The latter form a *σ*-*π* interaction with the residue *V*64, a non-conventional hydrogen bond with the residue *P*83 and some non-polar contacts with the residues *L*128 and *D*85, while the former makes hydrogen bonds with *A*132 oxygen atom and *N*66 side-chain nitrogen, besides non-conventional hydrogen bonds with the side-chain of the residues *I*126 and *K*78.

The MFCC scheme yields not only the individual pembrolizumab/PD-1 interactions but also important information from the pair-interactions that could be otherwise missing, namely: *R*99_*HC*_-*D*85 (*ε*_20_: −9.75 kcal mol^−1^; *ε*_40_: −7.43 kcal mol^−1^), *Y*35_*HC*_-*S*87 (*ε*_20_: −2.13 kcal mol^−1^; *ε*_40_: −2.08 kcal mol^−1^), *M*105_*HC*_-*K*131 (*ε*_20_: −6.61 kcal mol^−1^; *ε*_40_: −6.06 kcal mol^−1^), and *D*108_*HC*_-*K*131 (*ε*_20_: −6.56 kcal mol^−1^; *ε*_40_: −4.82 kcal mol^−1^).

The residues *M*105_*HC*_ and *D*108_*HC*_ make hydrogen bonds with the residue *K*131. The main-chain oxygen carbonyl of *M*105_*HC*_ makes a direct hydrogen bonds with the charged amine of this lysine residue, while the residue D108 is engaged in a water-mediated hydrogen bond with the same molecular group, yet presenting its negatively charged carboxyl group (*D*108) in a closer distance to positively charged amine group (see Fig. 5f). The pair *Y*35_*HC*_-*S*87 binding energy is governed by a hydrogen bond formed between the hydroxyl group of this tyrosine residue (*Y*35_*HC*_) and the main-chain carbonyl from *S*87. The pair *R*99_*HC*_-*D*85 is described for some authors as the only salt bridge formed between the pembrolizumab drug and the PD-1 receptor^26,30^.

### Pembrolizumab light-chain /PD-1 receptor interaction energy

**Figure 6:**
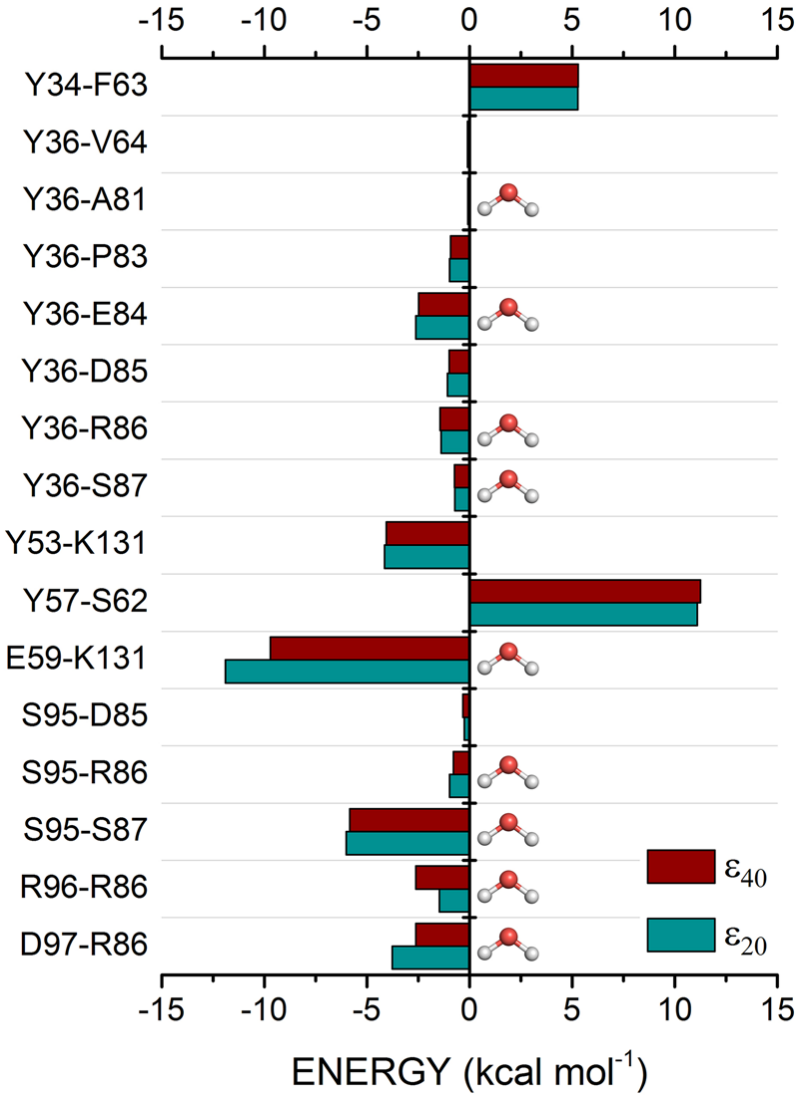
Graphical panel depicting the most relevant interactions involving the pembrolizumab light-chain residues *Y*34, *Y*36, *Y*53, *Y*57, *E*59, *E*95, *R*96 and *D*97.

The pembrolizumab light-chain is a little more distant of the PD-1 receptor than the heavy-chain one, as it can be inferred from its contribution to the total binding energy (*ε*_20_: −44.67 kcal mol^−1^; *ε*_40_: −47.75 kcal mol^−1^), as well as from the number of pairs formed with the receptor (148). This is consistent with previous crystallographic results^25,26^. According to Fig. 6, the most intense binding energies for the LC amino-acids is associated to the pair *E*59_*LC*_-*K*131 (*ε*_20_: −11.90 kcal mol^−1^; *ε*_40_: −9.70 kcal mol^−1^), being followed by the pairs *S*95_*LC*_-*S*87 (*ε*_20_: −6.00 kcal mol^−1^; *ε*_40_: −5.83 kcal mol^−1^), *Y*53_*LC*_-*K*131 (*ε*_20_: −4.15 kcal mol^−1^; *ε*_40_: −4.05 kcal mol^−1^), *R*96_*LC*_-*R*86 (*ε*_20_: −1.47 kcal mol^−1^; *ε*_40_: −2.62 kcal mol^−1^), and *Y*36_*LC*_-*E*84 (*ε*_20_: −2.62 kcal mol^−1^; *ε*_40_: −2.48 kcal mol^−1^).

**Figure 7:**
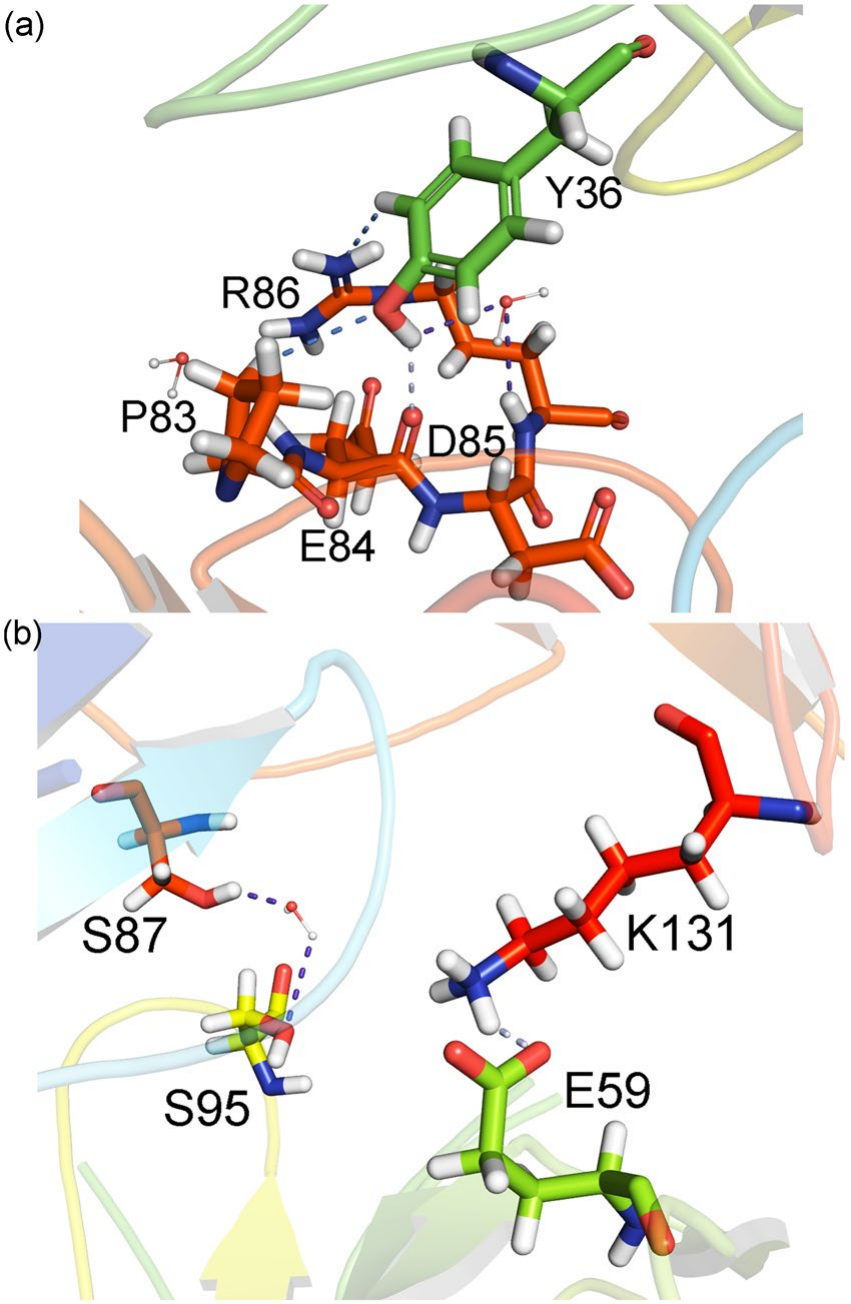
Intermolecular interactions of the most energetic pembrolizumab light-chain residues. (**a**) Interaction of the residue *Y*36 with the most relevant residues of the receptor PD-1. (**b**) The same for the residues *E*59 and *S*95. Dashed lines in light blue (purple) (marine) represent direct (water-mediated) (non-conventional) hydrogen bonds.

Figure 7 depicts some interactions made by the pembrolizumab light-chain residues linked to the PD-1 receptor. As one can see, the residue *Y*36_*LC*_ is surrounded by some charged residues (*E*84, *D*85 and *R*86) from the PD-1 receptor (Fig. 7a). However, it only makes a single direct hydrogen bond with the main-chain carbonyl of the residue *E*84, all the other interactions being non-conventional hydrogen bonds (*P*83 and *R*86), and water-mediated hydrogen bonds. It would be inconsistent with the position occupied by this residue among the most energetic one of the pembrolizumab light-chain, if the number of pairs formed with *Y*36_*LC*_ (10) would not overcome it. The residue making the biggest number of pair-interaction is *Y*34_*LC*_, namely 19. Notwithstanding, the residue *Y*34_*LC*_ shows a binding energy less than the *Y*36_*LC*_ one, due to an unfavorable interaction with the residue *F*63 (*ε*_20_: 5.27 kcal mol^−1^; *ε*_40_: 5.30 kcal mol^−1^).

Although only five pair-interactions were calculated with the residue *S*95_*LC*_ within a radius of 8.0 Å, it shows one of the highest interaction energies of the drug’s light-chain. Similar to the residue *Y*36_*LC*_, the majority of the surrounding residues make weak interactions, excluding *S*87 which forms a water-mediated hydrogen bond through the hydroxyl group from both side-chains (see Fig. 7b). It is also important to notice the attractive binding energy found to the *R*96_*LC*_-*R*86 pair, even with the positively charged guanidine group from both amino-acids, assuming a conformation where they are very close. These two arginine residues are in a T-shaped stacking interaction, which favors an attractive bind^30^. Finally, the *E*59_*LC*_-*K*131 pair forms the second salt bridge of the complex drug/receptor, depicting the highest individual interaction energy in the drug’s light-chain. This interaction is due to the negatively charged carboxyl group of the residue *E*59_*LC*_, and the positively charged amine of the residue *K*131 side-chain. It gives us the idea of the dynamic process which govern the interaction between the pembrolizumab Fab fragment and the extracellular region of the PD-1 receptor, such as the formation of a new salt bridge between the residues *D*108_*HC*_-*K*131 whose opposite charges from the side-chain are very close (3.6 Å).

**Figure 8:**
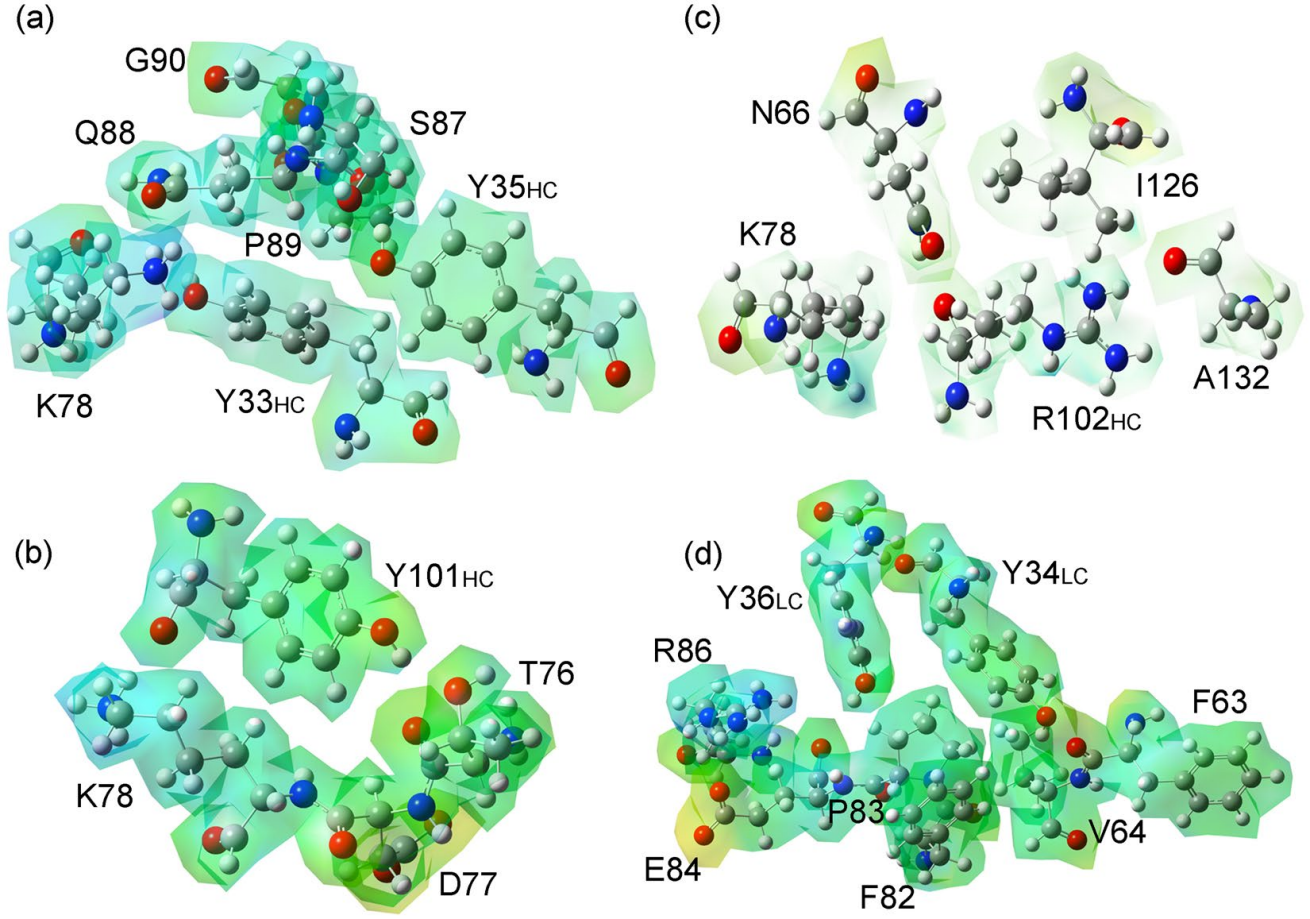
Electrostatic potential isosurfaces with the projected electron densities for some pembrolizumab residues interacting with the most attractive residues of the receptor PD-1.

For completeness, we display in Fig. 8 the electrostatic potential isosurface with projected electron densities for the pembrolizumab amino-acids bound to some of the most important residues at the binding pocket site.

### Total binding energy

**Figure 9:**
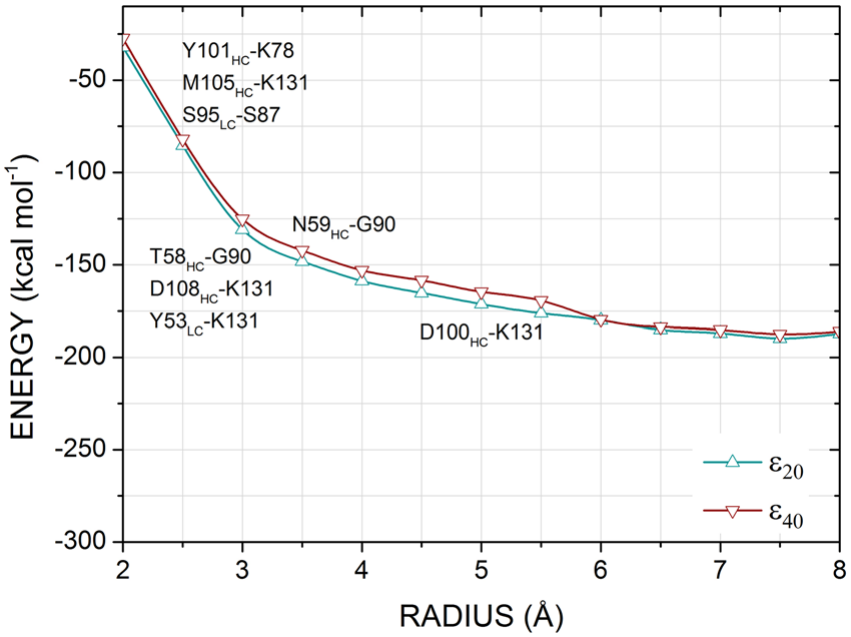
The total interaction energy as a function of the binding pocket radius *r* calculated using the GGA functional B97D. Pairs of amino-acids responsible for the regions of steepest negative and positive variation are highlighted.

To evaluate the binding interaction energies through fragment-based quantum mechanics method, it is important to take into account every significant attractive and repulsive amino-acid residue which can influence this mechanism. Therefore, instead of taking an arbitrary region of the binding site, we performed a search for an optimal binding pocket radius (*r*) in which a variation less than 10% of the sequential pocket radius could be observed after a radius increase. For this task, the binding pocket radius *r* is varied from 2.0 Å (−32.04 kcal mol^−1^ for *ε* = 20 and −27.29 kcal mol^−1^ for *ε* = 40) to 8.0 Å (−187.17 kcal mol^−1^ for *ε* = 20 and −186.08 kcal mol^−1^ for *ε* = 40) in order to determine the best value of *r*, found to be 6.0 Å corresponding to an energy of −179.94 (−179.60) kcal mol^−1^ for *ε* = 20 (40), from which the convergence was achieved (see Fig. 9). This result is in agreement with previous works, stating that after 6.0-7.0 Å the molecular interactions are weak^31,32^.

## Discussion

Cancer immunotherapy research is trying to overcome the cancer’s ability to resist the immune responses by stimulating the body’s own mechanisms to remain effective against the disease. Early clinical results using blocking agents against the human cell surface receptor PD-1 and its ligants PDL-1 and PDL-2, point to unprecedented rates of long lasting anti-tumor activity in patients with metastatic cancers of different histologies^33^. From the pharmaceutical point of view, however, the clinical development of the PD-1 pathway blockers requires an understanding of the signals that induce expression of its ligands within the tumor.

However, there are some patients showing resistance to the blockage of the receptor PD-1^34^. Over stimulation of immune responses, on the other hand, may lead to damage normal, healthy tissue. Therefore, to be more efficient, the design and production of such a pharmaceutical drug require a precise knowledge not only of its biochemical structure, but also its binding mechanism with the receptor PD-1, leading to a better understanding of how this complex influences the cancer tumor and its anti-angiogenesis therapies as well as its microenvironment. Computational tools based on quantum chemistry may be a promising step in this route, further increased by combination with other anti-cancer therapies.

Within this context, we presented here an *in silico* quantum biochemistry calculation of the electronic structure of the complex pembrolizumab Fab drug/PD-1 receptor in order to map its recognition surface, searching for the binding interactions that stabilize it. The quantum MFCC approach used here is a route to investigate accurately large biological systems with low computational cost, and has been applied previously to describe molecular interactions at the quantum level related to the collagen stability^35^, central nervous system disorders^36^, and breast cancer^37^, to cite just a few. There is no other quantum mechanical study relating the binding of the pembrolizumab drug at the PD-1 receptor, albeit its high pharmaceutical relevance.

After a convergence study on the size of the binding pocket sphere, we have considered all ligand-residue interactions within a pocket radius of 8.0 Å from the ligand. In addition, we have established the residues that play the most important roles on the binding affinity, namely: *Y*33_*HC*_ > *R*102_*HC*_ > *F*103_*HC*_ > *Y*101_*HC*_ > *E*59_*LC*_ > *M*105_*HC*_ > *N*52_*HC*_ > *Y*36_*LC*_ > *S*95_*LC*_ > *D*104_*HC*_. We also highlighted the energetic and structural differences between the pembrolizumab’s heavy-chain and light-chain besides unveiling the relevance to take into account the water molecules and weak interactions in the design of this therapeutic compounds. The strong attractive character observed in the amino-acid residues *Y*101_*HC*_, *R*102_*HC*_, *F*103_*HC*_, *D*104_*HC*_, *M*105_*HC*_ and *D*108_*HC*_ could give the base for the development of an improved drug targeting the PD-1/PD-L1 pathway by activating an immune resistance mechanism in response to endogenous anti-tumor activity, avoiding an immunosuppressive tumor microenvironment. It is also remarkable the relevance of the residues *K*78, *D*85, *P*89, *I*126 and *L*128 of the receptor PD-1. These amino-acids have been shown making pairs with almost all pembrolizumab residues, and mutational studies have displayed their relevance to the PD-1/ligand interaction^38^.

Some of these residues binding energies spectra are consistent with previous experimental studies^26,39^. Our results show also that in the Pembrolizumab/PD-1 complex, the residue D85 is associated with the strongest interaction energy regime, forming pairs with the residues *R*99_*HC*_ and *Y*36_*LC*_ through a salt bridge and hydrophobic contacts, respectively, while the residue R86 has no interaction whatsoever with any of the residues from the ligand, in agreement with the experimental results^30^. As shown by Fessas *et al*.^40^, one of the most relevant structural aspect of the Pembrolizumab/PD-1 complex is the interaction of the former with residues from the C’D loop of PD-1 (amino-acids P83-S93), emphasized in our previous section (Results), excluding the amino-acids *D*100_*HC*_, *Y*101_*HC*_, *Y*53_*LC*_, *Y*57_*LC*_ and *E*59_*LC*_, commonly showing one energetically relevant pair. These achievements confirm the relevance of this loop for anchoring Pembrolizumab and, likely, its ligand-based compounds.

Despite the absence of mutation experimental results, outcomes based on the crystallographic structures of the PD-1 protein mutants bound to the receptors PD-L1 and PD-L2 could be an useful background to understand the energetic behavior depicted in this work. Recently, Lázár-Molnár performed a mutagens study taking into account the wild-type human PD-1 receptor and a mutant form of the protein A132L^41^. Mutation of the amino-acids K78A in both proteins (wild-type and mutant) was responsible for the complete loss of the PD-L1 and PD-L2 interaction bind energy. Notwithstanding, K78, a residue from the C’ strand, is seen forming strong interaction pairs with a number of amino-acids from the Pembrolizumab, including the most energetic heavy-chain residues *Y*33_*HC*_, *D*100_*HC*_, *Y*101_*HC*_, *R*102_*HC*_ and *F*103_*HC*_. Also, although the amino-acid I126 makes non-conventional hydrogen bonds only with the heavy-chain residue *R*102_*HC*_, it is responsible for one of its strongest interaction energies. Meanwhile, the residues I134, L128 and E136, although not presenting a large number of energetically strong pairs, are related to *R*102_*HC*_ and *Y*103_*HC*_, which are the second and third most energetic Pembrolizumab heavy-chain residues, thus defining, along with the residue I126, a region of stabilization of the ligand. Taken together, it becomes clear the relevance of these residues for the inhibition mechanism of the Pembrolizumab.

To close this section, we would like to propose two pharmacophores that may be useful for the rational design of ligands targeting the receptor PD-1, looking for the development of antibody-based drugs. As one can note, the Pembrolizumab/PD-1 binding surface is mainly governed by a set of hydrophobic contacts strengthened by strong hydrogen bonds. Five residues from the PD-1 receptor can be observed to form two hot-spots required for the inhibition of the PD-1/PD-ligands. The first one is in the C’D loop and C’ sheet, while the second one is in the FG loop and F sheet, respectively. The former is composed by the residues K78, D85 and P89, with their most distinctive feature being the steric complementarity between the residue K78 and the hydrophobic/hydrophilic heavy-chain amino-acids *Y*33_*HC*_, *Y*101_*HC*_, *R*102_*HC*_ and *F*103_*HC*_. They are coupled to flexibilize the C’D loop, permitting the residue D85 to interact with the light-chain amino-acids *S*36_*LC*_ and *S*95_*LC*_. This pocket is ruled by hydrogen bonds and is, likely, one of the first docking points on the PD-1 surface due to its more external position, when compared to other motifs. The latter is located just nearby and has a most hydrophobic character, being composed by the residues I126 and L128. These residues are seen to accommodate the heavy-chain amino-acids *R*102_*HC*_ and *F*103_*HC*_ by anchoring them through hydrophobic interactions and non-conventional h-bonds. These proposed pharmacophores should provide a solid point for the design of new antibodies or small molecules targeting the receptor PD-1.

In summary, we have provided a quantum mechanics description of the Pembrolizumab/PD-1 complex at a molecular and energetic level by decomposing both proteins into amino-acid fragments and calculating the residue-residue interaction pair through an MFCC-based method within the density functional theory framework. The use of quantum mechanics in our calculations provided a good characterization of important molecular features by including an accurate electronic description and enhancing the differences of interaction intensity among the amino-acid pairs. Since Pembrolizumab has shown great improvement in the treatment of several cancer types, our results could favor the development of new compounds targeting the PD-1/PD-ligands, in special those based on the hot-spots, by avoiding structural features which are in minor relationship with this binding scenario. Notwithstanding, the quantum chemistry computational methods used in this work emerged as a simple and efficient alternative to unveil the drug’s amino-acids residues that play the most important role on the binding affinity of the receptor-ligand complex. No doubt, taking into account the small cost/benefit of the operation, *in silico* approach, not only in clinical oncology but also in pharmaceutical medicine in general, is an important initial step toward the development of efficient cancer pharmaceutical drugs.

## Methods

The calculations performed in this study have taken full advantage of the first X-ray crystal structure of human programed cell death protein 1 (PD-1) solved in complex with a Fab fragment of pembrolizumab (PDB ID: 5GGS) at 2.00 Å of resolution. The protonation state of the receptor PD-1 and its ligand was adjusted according to the results obtained from the PROPKA 3.1 package^42^. Afterwards, atoms not resolved by X-ray diffraction and therefore absent in the crystallographic files, as hydrogens and some amino-acid side-chains, were added to the structures and submitted to a classical geometry optimization fixing the other atoms. This optimization was performed using the classical force field CHARMm22 (Chemistry at Harvard Molecular Mechanics 22 forcefield), which is especially parametrized for organic molecules^43^. The calculations were carried out with convergence tolerances set to 10^−5^ kcal mol^−1^ (total energy variation), 0.001 kcal mol^−1^Å^−1^ (RMS gradient) and 10^−5^ Å (maximum atomic displacement).

The interaction energy between the amino-acids from the pembrolizumab (at position R_*i*_) and PD-1 (at position R_*j*_) was calculated by using the formalism of MFCC fragmentation scheme proposed by Zhang and Zhang^44,45^, and modified by Rodrigues and co-workers to calculate protein-protein interactions^28^, within a DFT framework. For each amino acid residue of interest at position R_*i*_, we draw an imaginary sphere with radius equal to 8.0 Å, and evaluate the interaction energy EI(R_*i*_-R_*j*_) with each residue at position R_*j*_, considering at least one atom inside the sphere. Hence, the scheme to compute the residue-residue interaction energies follows the MFCC approach, in which the interaction energy between the residue R_*i*_ and the other residues R_*j*_ was calculated according to:

1 $$\begin{array}{rcl}EI({R}_{i}-{R}_{j}) & = & E({C}_{i}{R}_{i}{C}_{i\ast }-{C}_{j}{R}_{j}{C}_{j\ast })-E({C}_{i}{R}_{i}{C}_{i\ast }-{C}_{j}{C}_{j\ast })\\  &  & -E({C}_{i}{C}_{i\ast }-{C}_{j}{R}_{j}{C}_{j\ast })+E({C}_{i}{C}_{i\ast }-{C}_{j}{C}_{j\ast })\end{array}$$

where the C_*i*_ and C_i*_ (C_*j*_ and C_j*_) caps are respectively the neighbour residues R_*i*−1_ and R_*i*+1_ (R_*j*−1_ and R_*j*+1_) attached to the reference residue R_*i*_ (R_*j*_). The term E(C_*i*_ R_*i*_ C_i*_ − C_*j*_ R_*j*_ C_j*_) corresponds to the total energy of the fragment comprises both residues as well as their capped residues. The second (third) term, E(C_*i*_ R_*i*_ C_i*_ − C_*j*_ C_j*_) [E(C_*i*_ C_i*_ − C_*j*_ R_*j*_ C_j*_)], gives the total energy of the system formed by the capped residue R_*i*_ (R_*j*_) and the hydrogenated caps of R_*j*_ (R_*i*_). E(C_*i*_ C_i*_ − C_*j*_ C_j*_) is the total energy of the system formed by the caps only.

In order to achieve the structural stability of the complex promoted by interactions with extended hydration network, all water molecules forming hydrogen bonds with a particular residue were included for completeness in the fragments. The analysis of the binding scenario were based in the criteria adopted by Bezerra *et al*.^35^.

Energetic calculations for each residue were performed using the Gaussian G09 code^46^, within the density functional theory (DFT) formalism, with the generalized gradient approximation (GGA) functional B97D (for a review see ref.^47^). It was proposed by Grimme^48^ and include dispersion terms in order to improve the description of the non-covalent interactions, being recently used together with the D3 dispersion correction under vacuum and COSMO continuum solvation model to calculate the binding energy of some protein ligand complexes within a MFCC scheme^49^. We selected the 6–311 + G(d,p) basis set for the calculations of the expanded Kohn-Sham orbitals for all electrons.

The representation of molecular environment is a necessary step for the theoretical study of molecular properties. Recently, models of implicit solvation have been used in the MFCC scheme by Ourique *et al*.^50^ and Dantas *et al*.^51^ to calculate binding affinity in protein-ligand complexes, by assuming different values of the dielectric constant (*ε*) to take into account the effect of the protein and solvent environment in the evaluation of the electrostatic energies. Here, we have employed the MFCC approach together with the CPCM (Conductor-like Polarizable Continuum Model) continuum solvation model^52^ with dielectric constants *ε* considered to be 20 (*ε*_20_ and 40 (*ε*_40_), respectively) to increase the similarity with the protein environment and estimate energy effects, such as the electrostatic polarization promoted by the solvent.

### Data availability

All other relevant data are available from the corresponding authors upon reasonable request.
